# Perceived impact of COVID-19 on routine care of patients with chronic non-communicable diseases: a cross-sectional study

**DOI:** 10.11604/pamj.2022.43.212.32769

**Published:** 2022-12-30

**Authors:** Desalew Tilahun Beyene, Kenenisa Tegenu Lemma, Samira Awel Sultan, Ismael Ahmed Sinbiro

**Affiliations:** 1 Department of Adult Health Nursing, School of Nursing, Institute of Health Sciences, Jimma University, Jimma, Ethiopia,; 2 Department of Pediatrics and Child Health Nursing, School of Nursing, Institute of Health Sciences, Jimma University, Jimma, Ethiopia,; 3 Department of Maternity Health Nursing, School of Nursing, Institute of Health Sciences, Jimma University, Jimma, Ethiopia

**Keywords:** Perceived-impact, COVID-19, routine care, chronic diseases, Ethiopia

## Abstract

**Introduction:**

patients with chronic non-communicable diseases (chronic liver diseases, chronic respiratory diseases, neurologic diseases, chronic kidney diseases, cardiovascular diseases, diabetes mellitus, and hypertension), primarily poor, rural and neglected populations, have had difficulty accessing health care and have been severely impacted both socially and financially in during the pandemic. As a result, this study was designed to assess the perceived impact of COVID-19 on routine care of chronic non-communicable disease patients in Ethiopia.

**Methods:**

a cross-section survey was conducted among 404 participants from April 1^st^ 2021 to May 30^th^ 2021. Data were collected via interviewer administered questionnaires administered by pre-tested interviewers on socio-demographic characteristics, treatment and clinical features and routine care questionnaires that have been adapted and modified from different literatures. The study consisted of all adult outpatients with at least one chronic non-communicable disease who were followed up. Data were analyzed using the Statistical Package for Social Sciences Version 23.

**Results:**

of the 422 participants, 404 responded for a response rate of 95.7%. One out of two (203, 50.2%) participants was aged 40 to 50 years. Ninety-one out of hundred (367, 90.8%) participants continued to receive routine care face-to-face during COVID-19. One-third (141, 34.9%) of study participants had good management of the chronic non-communicable diseases care in the middle of pandemic. A total of 167(41.34%) participants thought they were moderately affected changes in healthcare services since the COVID-19 outbreak. Nearly one-third (130, 32.2%) of participants were sometimes affected by medication shortages since the start of COVID-19.

**Conclusion:**

this study highlights that most participants continued to receive routine care face-to-face during the COVID-19. About forty-one out of 100 participants perceived that they were moderately affected changes in healthcare services since the outbreak of COVID-19. One-third of participants sometimes perceived that they were affected by medication shortages since the start of COVID-19.

## Introduction

The coronavirus (COVID-19) pandemic began in Wuhan, China at the end of 2019 and spread to a dozen countries in early 2020. Since then, the number of cases has continued to rise exponentially throughout the world [1], including in Ethiopia. Today, COVID-19 is a global pandemic with unprecedented health, economic and social consequences for countries around the world [2]. In the past few years, the COVID-19 outbreak has significantly changed the rhythm of human life and overwhelmed the healthcare systems of many countries including developed countries ) [1].

Ethiopia experienced its first case of COVID-19 in March 2020. The infection has spread across all regions of the country. In response, the government has taken a number of actions to prevent the spread of this disease. Locking down all schools, declaring social distancing and hand hygiene, restricting large gatherings, limiting travel, preparing health facilities for treatment and quarantining individuals who had known contact and travel history for 5 days were some of the actions the Ethiopian government took [3]. This in turn worsens the health care system, standard of living and cultural norms of people in developing countries such as Ethiopia. Reallocating funds to fight COVID-19 could undermine gains already made to control non-communicable diseases [4]. Routine chronic non-communicable diseases care represents a significant challenge during this pandemic. Continuing routine care for people with chronic non-communicable diseases despite the pandemic is important to prevent long-term impacts on health and mortality [5].

The COVID-19 pandemic has prompted the world to explore innovative ways of continuing outpatient care [3] including case identification, contact tracing, isolation and quarantine, which are actions taken to control the spread of the disease in addition to the preventive measures put in place mainly promoting social distancing and sanitary measures [2]. In developed countries many patients with chronic non-communicable diseases can communicate with their own health care professionals especially during this pandemic through internet-based programs and applications (telemedicine), unlike developing countries such as Ethiopia. Not only that internet is not available to many parts of the country, but even in places that have internet connectivity the people may lack internet literacy. In addition, there is a lack of data on the perceived impact of the pandemic on the routine care of patients with chronic conditions in healthcare settings in the current study area. As a result, this study aimed to assess the impact of the pandemic on the routine care of patients with chronic conditions at the public hospital in the Jimma area of Ethiopia.

## Methods

**Study area and period:** the study was conducted in public hospitals in Jimma zone, south-west Ethiopia from April 1^st^ 2021 to May 30^th^ 2021. Jimma zone is located 350 km from Addis Ababa. The zone is divided into 18 districts and a town administration. There are eight hospitals (one referral and teaching hospital, one general hospital and six primary hospitals), 115 health centres and 520 health posts in Jimma zone. An estimated 15 million people from within the country as well as from border zones and South Sudan avail health services from eight public hospitals in Ethiopia, with Jimma Medical Center (JMC) being a major service provider. During the study period, there were a total of 5800 patients on follow-up in the four public hospitals in the South west of Ethiopia i.e. 2000 patients with chronic non-communicable diseases at JMC, 1300 at Agaro Hospital, 1000 Seka hospital and 1500 Shenen Gibe Hospital.

**Study design, source population and study population:** facility based cross-sectional study was used. All patients on chronic follow-up at public hospitals in the Jimma zone, southwest Ethiopia were used as the source population.

**Eligibility criteria:** all Adult outpatients with at least one chronic illness on chronic follow-up at public hospitals in Jimma zone who visited chronic follow-up during the study period were included and severely sick patients in need of immediate medical intervention were excluded.

**Sample size determination and sampling technique:** sample size was determined by using a single population proportion formula by considering a 50% proportion as there is no previous study. The formula for calculating the sample size (n) is as follows:


$$n=\frac{{\left(Z\alpha /2\right)}^{2}p\left(1-p\right)}{d^{2}}$$

Where, n= Minimum sample size, p= an estimate of the prevalence rate for the population, d= the margin of the sampling error and Z_β/2_= standard normal variance (1.96)^2^ is mostly 5%, that is with a 95% confidence level.

n = (1.96)^2^x0.5(1-0.5)/(0.05)^2^= 384

By considering 10% non-response rate the final sample size is 422.

**Figure 1:**
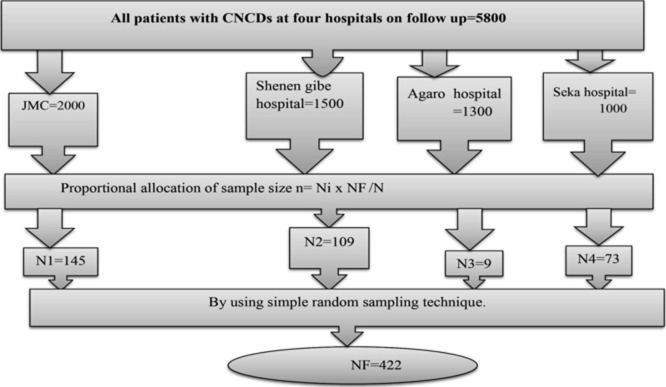
diagramatic representation of sampling procedure; where NF = final sample size (422), Ni = number of patience in each hospital and N = total number of patients in the time of data collection period in four hospitals chronic non-communicable disease (CNCD) = 5800

**Sampling procedures:** Figure 1 shows the sampling procedure. There were eight public hospitals in Jimma zone, and four hospitals from eight were selected using simple random sampling. Health posts and health centers were excluded because there was no chronic follow-up in the health posts and even though health centers had chronic follow-up, they had small number of patient flow as patients don't get adequate services they were referred to nearby hospital. The final sample size for the study was proportionally allocated to each selected hospital. An individual study participant was selected using systematic random sampling techniques from the chronic follow-up registration book of each hospital with a K-value=13.

**Data collection tools:** a pre-tested, structured interviewer-administered questionnaire was used for data collection which contained three parts including socio-demographic characteristics, clinical and treatment related characteristics and were used to assess the perceived impact of COVID-19 on routine care which was adapted and modified from different literature [3-6]. Perceived impact of COVID-19 was measured by 5-items [7] and each item score was summed up and finally described as described in Table 3. The instrument was translated to the Afaan Oromo and Amharic versions by experts who were fluent in both languages and back translated to English for consistency. The Afaan Oromo and Amharic versions questionnaire were used for the data collection.

**Data collection procedure:** interviewer-administered structured questionnaire and card review were used to collect data. Two Bachelor of Science degree (BSC) holder nurses and one Masters of Science (MSC) nurse were recruited as data collectors and supervisor respectively.

**Data quality control:** both data collectors and supervisor were trained for one day on the objectives of study and data collection techniques. A supervisor checked the completeness and consistency of the questionnaire. The principal investigator evaluated the data before analysis to verify the completeness of the collected data. The pre-test was performed with 5% of the sample size participants prior to actual data collection at the Limmu hospital chronic illness clinic follow up to assess the clarity and reliability of the data collection tool. Limmu hospital was chosen as the pilot study because it is far from the selected hospitals which in turn helps to prevent information dissemination/contamination and second it has similar sociocultural background participants with approximate patient flow and services in comparison with the study hospitals. Data from the pre-test were not included in the actual data. Scale reliability was assessed using the internal consistency of the reliability test. To date, all constructs of routine health care, (RHC) questionnaire reported excellent reliability with an overall alpha value of 0.90.

**Data processing and analysis:** the data were entered and coded in Epi-Data version 3.1. Data were exported to SPSS version 23 for analysis. Descriptive statistics were computed.

**Ethical consideration:** prior to data collection, ethical approval was obtained from the Institutional Review Board (IRB005/2021) of Jimma University and administrative permission was also obtained from JMC. During data collection, written informed consent was obtained from the literate participants and oral consent from the illiterate participants. The study participants were briefed on the study objectives and right to withdraw at any point. Data were collected anonymously.

**Operational definition**

***Chronic non-communicable diseases:*** (chronic liver diseases, chronic respiratory diseases, neurologic diseases, chronic kidney diseases, cardiovascular diseases, diabetes mellitus, and hypertension) [8].

***Perceived impact of COVID-19:*** was measured by 5-items [7] and each item score was summed up and finally described as described in the Table 3.

**Variables:** socio-demographic variables (age, sex, educational status, marital status, residence, occupational status and household monthly income) and clinical and treatment related characteristics (duration of treatment, presence of respiratory symptoms in the past 14 days, travel history to other areas during COVID-19 outbreak, contact history with a known COVID-19 case, source of medication and presence of comorbidity).

**Funding:** Jimma University covered only the survey cost for this study and there is no funding organization. This funding organization had no role in the design of the study, collection, analysis, and interpretation of data, or in writing the manuscript.

**Availability of data and materials:** due to the lack of consent from the study participants to disclose raw data, these data could not be made available to protect the participants' identities.

## Results

**Sample characteristics:** Table 1 presents the sample characteristics indicating that about half (50.2%) of the participants were in the age range of 40-50 years. More than half (57.4%) of participants were female. Only over one-third (39.6%) of the study participants had no education. More than three-forth (79.5%) of the participants were married and one in two (52.5%) of participants were urban residents.

**Table 1:** socio-demographic characteristics of the study sample

Variable	category	Frequency	percent
Age	18-39 years old	99	24.5
	40-59 years old	203	50.2
	≥60 years	102	25.2
Sex	Female	232	57.4
	Male	172	42.6
Educational status	None	160	39.6
	Primary or 1-8 grades	141	34.9
	Secondary and higher	103	25.5
Marital status	Married	321	79.5
	Single	49	12.1
	Widowed	32	7.9
	Divorced	2	0.5
Residence	Urban	212	52.5
	Rural	192	47.5
Occupational status	Famer	122	30.2
	Housewife	119	29.5
	Government employee	83	20.5
	Merchant	56	13.9
	Labor worker	24	5.9
Household monthly income	<500 Birr	81	20.0
	500-1000 Birr	53	13.1
	>1000 Birr	270	66.8

**Clinical and treatment related characteristic:** Table 2 summarizes clinical and treatment related characteristics of the participants. More than half (58.9%) of them had been on treatment for less than five years Most (80.2%) participants did not have a contact history with a known COVID-19 case.

**Table 2:** clinical and treatment-related characteristics

Variable	category	Frequency	percent
Duration of treatment	<5 Years	238	58.9
	5-10 Years	94	23.3
	>10 Years	72	17.8
Presence of respiratory symptoms in the past 14 days	Yes	80	19.8
	No	324	80.2
Travel history to other areas since your last visit	Yes	34	8.4
	No	370	91.6
Contact history with a known COVID-19 case	Yes	46	11.4
	No	358	88.6
Source of medication	Free	119	29.5
	Payments	285	70.5
Presence of comorbidity	Yes	142	35.1
	No	262	64.9

**Figure 2:**
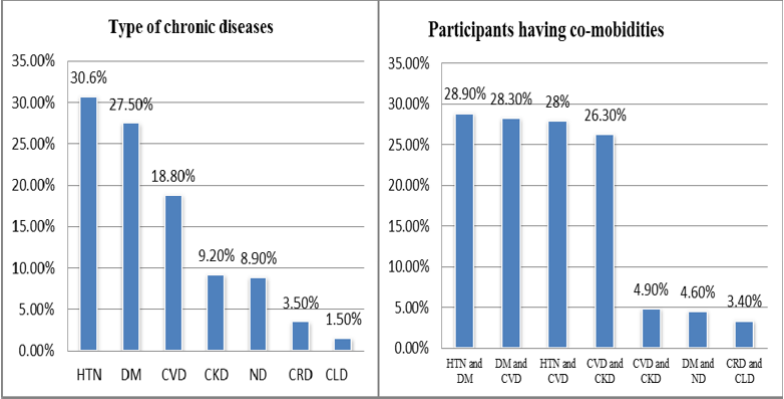
chronic non-communicable disease and comorbidities most affected by COVID-19

**Routine Health Care (RHC):** Table 3 describes the participants' routine healthcare services. Ninety-one out of hundred (90.8%) participants continued to receive routine care face-to-face during COVID-19. Only more than one-third (34.9%) of the study subjects had poor management of the chronic disease care since the outbreak of COVID-19 while the majority (41.34%) participants thought they were moderately affected by changes in healthcare services since the outbreak of COVID-19. Just one third of participants were sometimes impacted by medication shortages since the start of COVID-19. More than half of participants reported that their health condition was worsened since the outbreak of the COVID-19. Figure 2 demonstrates chronic non-communicable diseases and comorbidities most affected by COVID-19. Over a quarter of the patients had hypertension. Most participants had at least one co-morbidity.

**Table 3:** routine health care as perceived by the participants

Routine health care questions	Responses	Frequency	Percent
How are you continuing to get routine care during COVID-19?	Face-to-face	367	90.8
	Telephone	10	2.5
	Both (face-to-face and telephone)	25	6.2
	Othera	2	0.5
How has the management of chronic disease care for you been since the outbreak of COVID-19?	Very poor	72	17.8
	Poor	14131	34.97.7
	Fair	76	18.8
	Good	31141	7.734.9
	Excellent	84	20.8
What effect do you think changes in healthcare services have had on you since the outbreak of COVID-19?	No effect	50	12.38
	Mild effect	106	26.24
	Moderate effect	167	41.34
	Severe effect	81	20.04
How frequently you impacted by medication shortages since the start of COVID-19?	Never	49	12.1
	Rarely	61	15.1
	Sometimes	130	32.2
	Often	84	20.8
	Always	80	19.8
Has your health condition worsened since the outbreak of COVID-19?	Yes	250	61.9
	No	154	38.1

a- Facebook messenger.

## Discussion

COVID-19 is a global public health emergency unprecedented in modern history [9] that has had direct and indirect effects on people with chronic non-communicable diseases. In addition to morbidity and mortality, high rates of community spread and various mitigation efforts, including stay-at-home recommendations, had disrupted lives and created social and economic hardship [10]. It is worthwhile that routine care continues despite the pandemic, to avoid a rise in non-COVID-19 related deaths and morbidity [3].

However, there is a paucity of information on perceived impact of COVID-19 on routine care for people with chronic conditions in the study area. Thus, to our knowledge, this is the first study in the area. Overall, most participants continued to receive routine care face-to face during COVID-19 which is higher than study conducted in United Kingdom [3]. This discrepancy might be due to most participants had no education which deprived them of opportunities to undergo follow-up via phone calls, telemedicine, videos and other social media which in turn imposes them to get routine care face-to-face which is risky to contract COVID-19.

Over one-third of study participants had poor management of chronic non-communicable diseases care since the COVID-19 outbreak. This means that the pandemic diverted the attention of world to adapting to new ways of delivering care using telemedicine to reduce face-to-face contact. Adapting new ways of virtual healthcare and digital technologies is imperative to allow HCPs to continue routine appointments and the use of apps can support the self-management of chronic conditions. Nonetheless, most people with non-communicable diseases live in low-middle income countries, where these technologies may not be widely available or practical [3] including Ethiopia. Furthermore, those with comorbidities may depend heavily on regular check-ups or hospital appointments to manage risk factors or experience delays in treatment which may potentially have severe consequences [3].

However, majority participants thought they were moderately affected by changes in healthcare services since COVID-19 outbreak. People with chronic conditions are not only affected by the COVID-19 pandemic in a direct manner, but also in an indirect manner, that is, People with chronic conditions focus more on contracting infection than the existing disease. The COVID-19 pandemic has disrupted all societies, including routine health care systems [11]. Resources at all levels shifted away from chronic disease management and prevention during the outbreak. This leads to serious concerns about the indirect health impact of COVID-19, especially on chronic non-communicable diseases with increased complications and augmented progression due to delayed and diminished access to care and disruption in follow-up at the primary care level [11].

One-third of the participants were sometimes affected due to medication shortages since the start of COVID-19. Because of its high transmissibility and capacity to disrupt international travel and business, the pandemic created travel and business restrictions and was responsible for [12] impact on people with chronic conditions by medication shortages. This might also be because resource allocation at all levels has shifted away from chronic disease management and prevention during the outbreak [11].

More than half of the participants reported that their health had worsened since the start of the COVID-19 pandemic. This means that the pandemic has put patients with chronic conditions in complications and difficulties in routine medical care due to delayed transportation, shortage of medications, and human resources are among the contributing factors for patients with chronic non-communicable diseases to suffer from worsened health conditions during infectious disease pandemic [13]. The study was limited to and only included those who were on follow-up. The study is also limited to descriptive analysis; thus, rigorous analysis was not conducted to control for confounding variables, and the association between outcome and predictors was not done because of its descriptive nature as indicated in the prior study. As this was a single center cross-sectional study without a robust analysis, the generalizability of study is questionable.

## Conclusion

In conclusion, people with chronic conditions in low income countries including Ethiopia are disproportionately affected by the pandemic as they continue to receive face-to-face routine care. Thus, healthcare providers (HCP) should provide meticulous care to patients who receive routine care face-to-face to curb the transmission of the pandemic and manage the existing chronic condition. On the top of this, HCP should offer all inclusive patient centered care for all patients not only to treat the pandemic but also to tackle chronic conditions related comorbidity, mortality and complications.

### What is known about this topic

- *Nowadays, COVID-19 is a global pandemic with unprecedented medical, economic and social consequences affecting nations across the world including Ethiopia;*
- *In developed countries many patients with chronic non-communicable diseases can communicate with their own health care professionals especially during this pandemic through internet-based programs and applications (telemedicine), unlike for developing countries like such as Ethiopia, Ethiopia;*
- *Routine care for chronic disease is an ongoing major challenge during pandemic*.

### What this study adds

- *In our study, most participants continued to receive routine care face-to-face during COVID-19;*
- *One-third of the patients reported being inconvenienced due to shortage of medications;*
- *Care was unaffected for every one in three patients*.

## References

[1] Guo D, Han B, Lu Y, Lv C, Fang X, Zhang Z (2020). Influence of the COVID-19 Pandemic on Quality of Life of Patients with Parkinson´s Disease. Parkinsons Dis.

[2] Haftom M, Petrucka P, Gemechu K, Mamo H, Tsegay T, Amare E (2020). Knowledge, Attitudes, and Practices Towards COVID-19 Pandemic Among Quarantined Adults in Tigrai Region, Ethiopia. Infect Drug Resist.

[3] Chudasama YV, Gillies CL, Zaccardi F, Coles B, Davies MJ, Seidu S (2020). Impact of COVID-19 on routine care for chronic non-communicable diseases: A global survey of views from healthcare professionals. Diabetes Metab Syndr.

[4] Lim SL, Woo KL, Lim E, Ng F, Chan MY, Gandhi M (2020). Impact of COVID-19 on health-related quality of life in patients with cardiovascular disease: a multi-ethnic Asian study. Health Qual Life Outcomes.

[5] Schwartz MR, Oppold P (2020). The Impact of Assistive Technologies on Quality of Life and Psychosocial Well-Being. Psycho-Social Perspectives on Mental Health and Well-Being: IGI Global.

[6] Abuduxike G, Aşut Ö, Vaizoğlu SA, Cali S (2020). Health-seeking behaviors and its determinants: a facility-based cross-sectional study in the Turkish Republic of Northern Cyprus. Int J Health Policy Manag.

[7] Sahoo KC, Kanungo S, Mahapatra P, Pati S (2021). Non-communicable diseases care during COVID-19 pandemic: A mixed-method study in Khurda district of Odisha, India. Indian J Med Res.

[8] Organization WH WHO global coordination mechanism on the prevention and control of noncommunicable diseases: final report: WHO GCM. World Health Organization.

[9] Ornell F, Halpern SC, Kessler FHP, Narvaez JCdM (2020). The impact of the COVID-19 pandemic on the mental health of healthcare professionals. Cad Saude Publica.

[10] Hacker KA, Briss PA, Richardson L, Wright J, Petersen R (2021). Peer Reviewed: COVID-19 and Chronic Disease: The Impact Now and in the Future. Prev Chronic Dis.

[11] Danhieux K, Buffel V, Pairon A, Benkheil A, Remmen R, Wouters E (2020). The impact of COVID-19 on chronic care according to providers: a qualitative study among primary care practices in Belgium. BMC Fam Pract.

[12] Amimo F, Lambert B, Magit A, Hashizume M (2021). A review of prospective pathways and impacts of COVID-19 on the accessibility, safety, quality, and affordability of essential medicines and vaccines for universal health coverage in Africa. Global Health.

[13] Mohammedhussein M, Hajure M, Ebrahim J, Dule A (2021). Posttraumatic Stress Symptoms Among Patients with Chronic Medical Disease Amid Covid-19 Pandemic in Southwest Ethiopia. International Journal of Biomedical Engineering and Clinical Science.

